# Assessing change in the behavior of children and adolescents in youth welfare institutions using goal attainment scaling

**DOI:** 10.1186/1753-2000-7-33

**Published:** 2013-09-13

**Authors:** Rita Kleinrahm, Ferdinand Keller, Kerstin Lutz, Michael Kölch, Jörg M Fegert

**Affiliations:** 1 Department of Child and Adolescent Psychiatry and Psychotherapy, University Hospital Ulm, Steinhoevelstr 5, 89075 Ulm, Germany; 2 Vivantes Hospital, Landsberger Allee 49, 10249 Berlin, Germany

**Keywords:** Youth welfare institutions, Goal attainment scaling, Child behavior checklist

## Abstract

**Background:**

Evaluating youth welfare services is vital, both because of the considerable influence they have on the development of children and adolescents, as well as owing to the extensive financial costs involved, especially for child residential care. In this naturalistic study we have undertaken to evaluate changes in various behaviors of young people who are in youth welfare institutions, not only by using standardized questionnaires, but also specifically modified goal attainment scales (GAS). These scales were meant to represent the pedagogical objectives of youth welfare professionals as well as the individual goals of the young people in care.

**Methods:**

Goal attainment scales were used to ascertain behavioral changes in 433 children and adolescents (age 6 to 18 years) in 25 youth welfare institutions (day care and residential care) in Germany. Social and individual goals were rated by young people and caregivers together on at least two occasions. In addition, to examine potential problems of children and adolescents, quality of life as well as mental health and behavior problems were identified by the caregiver and also by the youth using a self-report inventory.

**Results:**

Many of the children and adolescents had experienced critical life events, problems in school, impaired quality of life, along with mental health and behavior problems (range: 41-87%). During their stay in day care or residential care institutions, children and adolescents showed some improvement in social goals (Cohen’s d = 0.14-0.44), especially those young people with deficits at the beginning, and with regard to mental health and problem behavior (d = 0.10-0.31). For individual goals, progress was even more pronounced (d = 0.75). Improvements to social goals were more pronounced if mental health and behavior problems decreased. This link to changes in behavioral and emotional problems was only ascertained to a limited extent for individual goals.

**Conclusions:**

Young people residing in youth welfare institutions achieved individual and social goals and improved with regard to behavior problems. The applied goal attainment scales are well suited for measuring individual change in children and adolescents and constitute a relevant addition to established instruments. Furthermore, their advantages include cooperative goal setting, the assessment of goals by caregivers and young people, and congruence with the pedagogical objectives of professionals.

## Background

Evaluating therapeutic and social interventions is essential in research and clinical practice, in youth welfare services, and also as the basis for policy decision-making in health and social departments. Quality development and assurance are mandatory in health care systems as well as in youth welfare institutions [1-3]. Of the three generally accepted aspects of quality – system structure, process and outcome – the importance of outcome quality is highlighted by the enormous financial costs of health care and youth welfare services [4-6]. Funding through public means is only justified if the intervention in question is effective and efficient. Moreover, in the youth welfare system, there is an ethical obligation to improve the living conditions of young people who need their support. Nevertheless, youth welfare services often disregard outcome quality [7].

To ensure quality, it is necessary to have objective, reliable, and valid measurements of all important outcome variables, such as symptom reduction, prevention of multiple placements, a means to participate in social life, development of relevant skills (e.g. social competence, adept handling of sickness, school performance), and the extent to which general therapeutic, pedagogical, or individual goals are reached. Symptom reduction is often the main outcome variable assessed; indeed, standardized psychometric measurements such as questionnaires about emotional and behavioral problems for patients and parents are important for determining the effectiveness of psychotherapy and youth welfare services. This is particularly true when considering the high prevalence of mental disorders and behavior problems found among adolescents in youth welfare institutions [8-12] and their effect on placement changes [13,14]. For example Burns, Phillips, Wagner, Barth, Kolko, Campbell & Landsverk [15] reported that 88.6% of the children and adolescents in group homes had CBCL total scores in the clinical range. In a study by Schmid, Goldbeck, Nützel & Fegert [16] 72.1% of the children in residential care had overall CBCL scores in the borderline clinical or clinical range.

In psychological and pedagogical contexts, there is an additional emphasis on client-reported outcomes. The standardized questionnaires mentioned above should be complemented in those contexts by instruments that are consistent with the widely used strengths-based approach in social work practice and that account for individual differences between clients [17-19]. Furthermore, they should support client participation, which is one of the key indicators for success in youth welfare services [19] and required by the United Nations Convention on the Rights of the Child [20] as well as the new German law to improve protection of children and adolescents [21]. Finally, these additional instruments should be sensitive to individual changes in target behaviors and measure the success with which individually defined goals are achieved [22].

A widely used technique for measuring individual changes is the so-called goal attainment scaling (GAS), which was developed by Kiresuk and Sherman [23] in community mental health services. Since then, it has been adapted for use in various settings, including social work practice [24-26], child psychology [27-29], psychotherapy [30], health promotion [31], occupational therapy [32], and pediatric rehabilitation [33]. GAS involves the following steps [34]: identifying the main issues of the client, translating these problems into at least three explicit and realistic goals, selecting a specific indicator for progress with regard to each goal, defining and reviewing the expected level of outcome, and specifying what constitutes a level of outcome that is somewhat higher and somewhat lower than expected as well as much higher and much lower than expected. The most effective way to set realistic, desirable individual goals is to negotiate and define them in cooperation with the client [35]. After a predefined time interval, the therapist / social worker and/or the client rates the actual outcome using this scale to measure the extent of individual change.

Psychometric properties were evaluated in reviews of goal attainment scaling in various research areas [33,36,37]: Reliability was found to be good (ICC = .88 - .93). Validity was demonstrated in several studies, but since GAS can be used in very different contexts, this suggests that it should be assessed anew on a case-by-case basis [36]. Sufficient sensitivity to measure individual progress in clients was shown by various studies as well.

Several advantages of using GAS were stated in the studies mentioned above: (1) reinforcement of client self-efficacy and motivation by emphasizing their success in reaching essential goals; (2) assessment of the critical target outcomes of a specific intervention instead of more general changes thanks to standardized questionnaires and the measurement of individual growth in individually relevant areas; (3) tendency to prevent frustration in both clients and interventionists because of its sensitivity to small, yet relevant changes; (4) increased intervention focus by accurately defining goals; (5) easy application in various fields, such as with children, adolescents, adults, and elderly people.

In Germany, GAS was used in youth welfare studies several times over the last decade. In a large prospective study financed by the Federal Ministry of Family Affairs, Senior Citizens, Women and Youth (JES study), a simplified version of GAS was used to estimate the percentage of goal attainment for goals of children or adolescents and their parents [38]. In the participating institutions, pedagogical practitioners predicted and rated goal attainment in three problem areas that appeared to be most significant. A similar procedure was used in a study called EVAS, in which instruments for performing checkups and evaluations in youth welfare institutions were developed [39]. A study of the organization of processes in youth welfare showed that the objectives of youth welfare services are often imprecisely defined, thus making evaluating the outcome quality all but impossible [40]. As a result, quality standards that include defining and validating goals as well as the responsibilities for achieving them were established [41]. As far as we know, GAS has not been used in other countries to evaluate youth welfare services in recent years.

In two studies, one in German youth welfare institutions [42] and one in a youth forensic context in Switzerland [43], GAS was modified and used to evaluate change in children and adolescents with respect to social and individual goal behavior during their stay. In these studies, professionals and clients rated goal attainment cooperatively using a computer-based tool. Current and intended behaviors in three relevant areas were defined together and reviewed after a predefined time interval, usually about every six months. This enabled children and adolescents to take part in the process of child services from the beginning. To increase the probability of goal attainment and improve its process, the necessary steps to get there were documented, and the responsibilities of the child/adolescent as well as the professional in charge were defined. In order to be able to compare clients, groups, or institutions in specific domains, Lutz, Kleinrahm, Kölch, Fegert & Keller [42] decided to measure not only individual goal attainment but also changes in the areas of social behavior that were generally important to young people (= social goals), such as integration in the peer group, behavior in school, social competencies, and practical skills [44].

In the current study, we established an Internet-based instrument as a standard evaluation tool in youth welfare institutions (day care and residential care) that incorporates social and individual goal attainment scales. The following questions were addressed:

(1) How do mental health / behavior problems and the social behavior of children and adolescents change while they are receiving youth welfare services?

(2) To what extent do children and adolescents achieve individual goals while they are receiving youth welfare services? And which topics are frequently represented in these individual goals?

(3) How do changes in social and individual goals relate to changes in mental health and behavior problems?

## Methods

### Procedure

An Internet-based instrument developed in cooperation with youth welfare professionals (CJD; Christian Association of Youth Villages [45]) and a software company (arielgrafik [46]) was introduced as a standard evaluation tool in 25 day care and residential care institutions of a large youth welfare organization in Germany. Professionals (social workers, caregivers, psychologists, educators) were trained in using the computer-based tool and asked to complete the questionnaires at the beginning of youth welfare services. Children and adolescents were shown by their guardians how to complete the self-report versions. Professionals and young people worked together to set goals at the beginning and rated change in goal behavior about every six months, depending on the procedures of the respective youth welfare service. Follow-up measures with questionnaires (by caregiver and self-report) were performed after the same time interval as that of goal attainment scaling.

Since this was a naturalistic study, time intervals between the beginning of services, initial measurement, and follow-ups, as well as the duration of youth welfare services varied from client to client. Moreover, not every instrument was used with every client. The following analyses show the results for all clients with individual goal attainment scores and, where available, the outcomes concerning social goals as well as mental health or behavior problems. Goal attainment and changes in mental health and behavior problems were calculated by the differences between the initial measurement (t1) and last available follow-up (tn).

### Instruments

The instrument contains two goal attainment scales developed in earlier studies [42,47]. One scale measures the attainment of individual goals, while the other measures changes in areas of social behavior that are important to most children and adolescents (see Table 1). These generally applicable goals were derived from a Delphi method [48] performed with professionals (social workers, psychologists, teachers, pedagogical practitioners, nurses) and adolescents in participating day care and residential care institutions. The chosen topics were expressed using eight social goals defined by the worst possible behavior (1) and the best possible behavior (7).

**Table 1:** **Social goals developed via the Delphi-method in a pilot study by Lutz, Kleinrahm, Kölch, Fegert** &**Keller**[42]

**Behavioral areas**	**Behavior axes**	**Goals**
Self-reliance	Autonomy	Independence
		Future perspective
	Contention	Conflict management
		Ability to criticize / take criticism
Social competence	Adaptation	Reliability / rule compliance
		Behavior at school / vocational training
	Affiliation	Integration into (peer)groups / friendship
		Ability to communicate

Goal attainment on both scales was recorded on a seven-point scale: Goal behavior is exhibited almost never (1), rarely (2), sometimes (3), occasionally (4), frequently (5), usually (6), always (7). In addition, the motivation of the client to change the targeted behavior is documented on a five-point scale ranging from ‘not motivated’ (1) to ‘very motivated’ (5).

In a pilot study, both goal attainment scales were found to be practical and methodically adequate [47]. Inter-rater reliability was good (ICC-coefficient: .68 - .88) with regard to social goals and even very good (ICC-coefficient: .90 - .96) for individual goals. Both scales were sensitive to changes with statistically significant t-values from 4.13 to 7.41 (p < .001) [42]. Construct validity was tested by means of correlations between goal attainment and the decrease of emotional and behavioral problems measured using the Child Behavior Checklist/4-18 (CBCL) [49].

The tool is supplemented by questions about the socioeconomic background, family history, school-related and health problems (basic documentary sheet based on the official German youth welfare statistics). Quality of life was determined using the Inventory for Assessing Quality of Life in Children and Adolescents [50]. There are two versions of this inventory, one for caregivers and one for children and adolescents. Seven items covering different aspects of quality of life are aggregated into a single problem score (0 = no problems, 7 = problems in all areas).

Moreover, mental health and behavior problems were assessed using standardized rating scales as well. The Child Behavior Checklist/4-18 (CBCL) [49] was completed by caregivers and the Youth Self-Report (YSR) [51] by the clients themselves. Both questionnaires comprise eight scales with 120 items: withdrawal, anxiety/depression, somatic complaints, social problems, thought problems, attention problems, delinquent behavior, and aggressive behavior. These scales can be combined into three broadband scores: internalizing behavior, externalizing behavior, and total problems.

### Statistical analyses

Changes in social and individual goals as well as in mental health and behavior problems were tested with t-tests for dependent variables. To describe the extent of change, Cohen’s d was used as a measure of effect sizes [52]. Correlations in between the social goals, between social goals and CBCL/YSR broadband scales as well as between the extent of change and the length of the time interval between measurements were tested using Pearson’s correlation coefficients. Changes in social and individual goals in relation to changes in mental health and behavior problems were tested my means of one-way analysis of variance (ANOVA), where changes in the CBCL total problem score was classified into four groups: (1) no (borderline) clinical behavior at the beginning as well as at the last measurement (T < 60 → T < 60), (2) problematic behavior at the beginning but not at the last measurement (T ≥ 60 → T < 60; decrease of problems), (3) no (borderline) clinical behavior at the beginning but at the last measurement (T < 60 → T ≥ 60; increase of problems) and (4) problematic behavior at the beginning as well as at the last measurement (T ≥ 60 → T ≥ 60). This categorization was chosen to illustrate how changes from clinically relevant CBCL scores to normal scores relate to changes in social goals. Cohen’s f was used as effect size [52]. Since converting CBCL scores into a categorical variable reduces the information contained, correlations between changes in goals and changes in CBCL/YSR were additionally tested using Pearson’s correlation coefficients. The level of significance was set at p < .01. To account for the large number of analyses, we adjusted p-levels using the Bonferroni correction. Effect sizes were calculated using MS Excel, while all other analyses were conducted with SAS 9.3.

## Results

### Participants

Caregivers (in our study they are invariably staff in the participating day care or residential care institutions) used the goal attainment scales with 433 children and adolescents from 2006 to 2010. Ages ranged from 6 to 18 years (M = 14.7, SD = 2.6). Girls (M = 15.5, SD = 1.9) were older than boys (M = 14.3, SD = 2.8; t = 5.52, df = 411.8, p < .001). The young people had been in their current institution for about 8 months before starting goal attainment within our project. Many of the children/adolescents experienced problems in their families, at school, or with regard to health issues (see Table 2). 86.8% indicated at least one critical life event in their past, with on average 3.3 (SD = 1.9) events being reported. Quality of life was rated as impaired by 41.5% of the young people, while caregivers considered it to be even up to 58.3% of the children and adolescents.

**Table 2:** Frequency in demographic variables and problem behavior

**N = 433**	**n**	**%**
Girls	157	36.3
Children lived with both their biological parents before placement.	123	28.4
Children did not live with any parent before placement.	128	29.6
Children lived in foster care or residential care directly before this placement.	67	15.5
Time lag between start of this placement and initial recording of individual goals (M = 8.0 months, SD = 11.5):		
< 1 month	92	21.2
1 to 3 months	106	24.5
3 to 12 months	138	31.9
> 12 months	97	22.4
At least one parent was not born in Germany (immigrant background).	76	17.6
Problems reported at school (M = 3.4, SD = 1.9)	365	84.3
Problem behavior (CBCL, T ≥ 60; caregiver report, N = 406):		
only internalizing	97	23.9
only externalizing	81	20.0
internal and external	122	30.1
Problem behavior (YSR, T ≥ 60; youth report, N = 398):		
only internalizing	75	18.8
only externalizing	61	15.3
internal and external	122	30.7
ICD-10 diagnosis (caregiver report)	112	25.9

CBCL = Child Behavior Checklist/4-18; YSR = Youth Self-Report.

Caregivers did not necessarily use all the instruments of the computer-based tool with every child or adolescent: Social goals were repeatedly assessed with 415 young people. Quality of life was evaluated with 429 children and adolescents. Mental health and behavior problems were rated for 406 young people in CBCL and by 398 adolescents in YSR. On average, individual and social goals were assessed 2.72 and 2.93 times respectively. The time lag between the first two measurements was about eight months (individual goals: M = 7.76, SD = 5.83; social goals: M = 8.08, SD = 5.80; CBCL: M = 7.75, SD = 5.16; YSR: M = 8.00, SD = 5.21).

### Change in mental health and behavior problems

At the initial measurement, caregivers as well as clients rated mental health and behavior problems of children and adolescents as borderline clinical on average (see Table 3). 56% showed less emotional and behavioral problems in caregiver reports after an average of 14 months, in youth self-reports, as many as 64% reported fewer problems. About 20% of the clients were rated as showing borderline clinical or clinical behavior at the beginning and subsequently improved to normal behavior (range: 16.1-21.5%). Internalizing behavior problems and overall problem behaviors in CBCL decreased over the course of time. Adolescents displayed significantly less problem behaviors on all broadband scales of YSR. Effect sizes, however, were small (d = 0.10 – d = 0.31). Correlations between the extent of changes in mental health and behavior problems and the time lapse between the initial and last measurements in both the caregiver and youth reports were negative, suggesting that the decrease of problem behavior was greater after a longer time interval. However, these correlations were not significant.

**Table 3:** Changes in mental health and behavior problems and social goals from initial to last measurement

	**M(t1)**	**M(tn)**	**SD(t1)**	**SD(tn)**	**t**	**df**	**p**	**Effect size**	**Improvement (%)**	**r(change, time)**
**Behavior problems**										
CBCL internalizing	60.75	58.62	9.94	10.49	3.98*	335	<.0001	0.21	19.1	-.09
CBCL externalizing	60.88	59.70	11.72	12.09	2.11	335	.0354	0.10	16.1	-.03
CBCL total problems	62.77	60.79	8.94	10.43	3.98*	335	<.0001	0.20	21.1	-.09
YSR internalizing	59.79	56.77	11.03	10.65	5.65*	316	<.0001	0.28	21.5	-.13
YSR externalizing	60.35	58.25	11.03	10.39	3.94*	316	<.0001	0.20	17.0	-.03
YSR total problems	63.01	59.79	10.70	9.94	6.44*	316	<.0001	0.31	20.8	-.10
**Social goals**										
Independence	4.32	4.89	1.32	1.27	8.69*	414	<.0001	0.44	53.7	.18*
Future perspective	4.28	4.65	1.55	1.58	4.49*	414	<.0001	0.24	45.1	.14
Conflict management	4.06	4.60	1.26	1.24	8.08*	414	<.0001	0.43	52.5	.05
Ability to criticize / take criticism	4.17	4.58	1.34	1.26	5.77*	414	<.0001	0.32	43.9	.07
Reliability / rule compliance	4.96	5.15	1.37	1.30	2.75	414	.0062	0.14	39.8	.06
Behavior at school / vocational training	4.61	4.86	1.55	1.42	2.95	414	.0034	0.17	41.4	.02
Integration into (peer)groups / friendship	4.80	5.29	1.45	1.35	6.92*	414	<.0001	0.35	47.5	.20*
Ability to communicate	4.60	4.98	1.15	1.11	6.25*	414	<.0001	0.34	46.5	.14
Total score	4.47	4.88	1.01	1.01	8.37*	414	<.0001	0.41	65.3	.16

CBCL = Child Behavior Checklist/4-18; YSR = Youth Self-Report; CBCL: N = 336, YSR: N = 317, social goals: N = 415; effect size = Cohen’s d: 0.2-0.5 small effect; improvement = percentage of clients whose assessment changed from T ≥ 60 to T < 60 in CBCL/YSR or who showed increase in this goal behavior; r = Pearson’s correlation between the extent of change and length of time interval between measurements: 0.1-0.3 small effect; p-level (adjusted) for 15 t-Tests: *p = .01 → p = .00067.

### Changes in social goals

At the initial measurement, all social goals were rated between “goal behavior is shown occasionally (4)” and “frequently (5)” on average. There were medium to high correlations in between the eight social goals at the initial as well as at the last measurement (see Table 4). Moreover, there were small to medium correlations between social goals and mental health / behavior problems (broadband scales of CBCL and YSR) at the initial measurement (see Table 5). Children’s and adolescents’ motivation to increase competencies ranged from 3.72 for “reliability / compliant to rules” to 4.11 for “independence”. Between 40% and 54% of the clients showed some improvement in social goals, and 65% showed overall improvement (see Table 3). On average, clients were rated significantly more competent in six domains over the course of time. There was no significant change in the goals “reliability / compliant to rules” and “behavior in school / vocational training” (both on the “adaptation” behavior axis). Effect sizes, however, were small (d = 0.14 - d = 0.44). The last column of Table 3 shows the correlations between the extent of changes in goal behavior and the duration between the initial and last measurements. There were significant correlations with regard to two of the goals, namely “independence” and “integration into (peer) groups / friendship”, with greater improvement after a longer time interval.

**Table 4:** Correlation matrix for social goals at the initial (t1) and last (tn) measurement

	**(1)**	**(2)**	**(3)**	**(4)**	**(5)**	**(6)**	**(7)**	**(8)**	**(9)**
**(1) Ability to communicate**		0.55*	0.51*	0.42*	0.51*	0.35*	0.46*	0.57*	0.72*
**(2) Conflict management**	0.64*		0.63*	0.51*	0.52*	0.45*	0.48*	0.51*	0.78*
**(3) Ability to criticize/take criticism**	0.60*	0.70*		0.49*	0.48*	0.43*	0.42*	0.47*	0.74*
**(4) Reliability/ rule compliance**	0.56*	0.59*	0.56*		0.49*	0.55*	0.40*	0.35*	0.72*
**(5) Independence**	0.61*	0.61*	0.56*	0.59*		0.46*	0.57*	0.52*	0.77*
**(6) Behavior at school/ vocational training**	0.46*	0.55*	0.52*	0.60*	0.49*		0.45*	0.35*	0.70*
**(7) Future perspective**	0.52*	0.56*	0.53*	0.52*	0.63*	0.50*		0.50*	0.74*
**(8) Integration into (peer)groups/ friendship**	0.54*	0.54*	0.47*	0.37*	0.46*	0.31*	0.47*		0.73*
**(9) Total score**	0.78*	0.84*	0.80*	0.78*	0.80*	0.72*	0.78*	0.67*	

Correlations were based on N = 431 at the initial measurement (t1; right triangle) and N = 415 at the last measurement (tn; left triangle); Pearson’s correlation coefficient r: 0.1-0.3 small effect, 0.3-0.5 medium effect, > 0.5 large effect; p-level (adjusted) for 72 correlations: *p = .01 → p = .000139.

**Table 5:** Correlation matrix between social goals and CBCL/YSR at the initial measurement

	**CBCL internalizing**	**CBCL externalizing**	**CBCL total problems**	**YSR internalizing**	**YSR externalizing**	**YSR total problems**
**(1) Ability to communicate**	−0.38*	−0.21*	−0.37*	−0.23*	−0.16	−0.21*
**(2) Conflict management**	−0.31*	−0.47*	−0.52*	−0.17	−0.34*	−0.28*
**(3) Ability to criticize/ take criticism**	−0.30*	−0.42*	−0.45*	−0.22*	−0.35*	−0.32*
**(4) Reliability/ rule compliance**	−0.14	−0.44*	−0.39*	−0.04	−0.31*	−0.19
**(5) Independence**	−0.28*	−0.29*	−0.42*	−0.22*	−0.28*	−0.29*
**(6) Behavior at school/ vocational training**	−0.11	−0.43*	−0.37*	−0.07	−0.38*	−0.26*
**(7) Future perspective**	−0.17	−0.26*	−0.31*	−0.16	−0.23*	−0.22*
**(8) Integration into (peer)groups/ friendship**	−0.35*	−0.29*	−0.44*	−0.26*	−0.21*	−0.24*
**(9) Total score**	−0.34*	−0.48*	−0.55*	−0.23*	−0.39*	−0.34*

Correlations were based on N = 404 for social goals*CBCL and N = 396 for social goals*YSR; Pearson’s correlation coefficient r: 0.1-0.3 small effect, 0.3-0.5 medium effect, > 0.5 large effect; p-level (adjusted) for 54 correlations: *p = .01 → p = .000185.

### Changes in social goals in relation to competencies at the initial measurement

The magnitude of changes in goal behavior differed in relation to the extent of competencies already exhibited at the beginning of child welfare. Children and adolescents whose social goal behaviors were rated low (total score < 4) at the beginning showed improvement more often (55% - 71%) than young people who were quite competent already (33% - 47%). Young people with deficits became more competent in all eight domains with medium to large effect sizes, whereas goal behaviors of already competent children and adolescents (total score ≥ 4) changed to a lesser degree with only small effect sizes (see Table 6).

**Table 6:** Changes in social goals from the initial to last measurement separated by competence at the beginning

	**M(t1)**	**M(tn)**	**SD(t1)**	**SD(tn)**	**t**	**df**	**p**	**Effect size**	**Improvement (%)**
**Low competence (total score < 4; N = 123)**									
Independence	3.06	4.20	0.98	1.15	10.05*	122	<.0001	1.07	69.1
Future perspective	2.88	3.85	1.25	1.65	6.15*	122	<.0001	0.66	60.2
Conflict management	2.91	4.02	0.86	1.25	8.86*	122	<.0001	1.03	71.5
Ability to criticize / take criticism	3.06	3.98	1.12	1.29	6.12*	122	<.0001	0.76	56.1
Reliability / rule compliance	3.75	4.44	1.22	1.34	4.85*	122	<.0001	0.54	55.3
Behavior at school / vocational training	3.26	4.32	1.36	1.49	6.17*	122	<.0001	0.74	61.0
Integration into (peer)groups/ friendship	3.49	4.66	1.24	1.54	8.00*	122	<.0001	0.84	65.0
Ability to communicate	3.62	4.48	0.87	1.20	7.73*	122	<.0001	0.82	61.0
Total score	3.25	4.24	0.51	1.03	10.48*	122	<.0001	1.22	81.3
**High competence (total score > =4; N = 292)**									
Independence	4.85	5.18	1.06	1.21	4.36*	291	<.0001	0.29	47.3
Future perspective	4.87	4.99	1.26	1.42	1.28	291	.2003	0.09	38.7
Conflict management	4.54	4.85	1.09	1.15	4.05*	291	<.0001	0.28	44.5
Ability to criticize / take criticism	4.64	4.84	1.13	1.16	2.60	291	.0098	0.17	38.7
Reliability / rule compliance	5.47	5.45	1.07	1.16	0.33	291	.7429	−0.02	33.2
Behavior at school / vocational training	5.18	5.09	1.25	1.32	0.99	291	.3224	−0.07	33.2
Integration into (peer)groups/ friendship	5.36	5.56	1.14	1.16	2.75	291	.0062	0.17	40.1
Ability to communicate	5.01	5.20	0.99	0.99	2.63	291	.0091	0.19	40.4
Total score	4.99	5.14	0.67	0.88	3.17	291	.0017	0.19	58.6

Effect size = Cohen’s d: 0.2-0.5 small effect, 0.5-0.8 medium effect, > 0.8 large effect; improvement = percentage of clients who showed increase in this goal behavior; p-level (adjusted) for 18 t-Tests: *p = .01 → p = .00056.

### Changes in individual goals

An average of 3.50 (SD = 1.81) goals were defined for each child/adolescent (range = 1–15). All in all, 1494 goals were rated. Clients exhibited improvement in 62% of the goals. However, only in 37% was the targeted characteristic met. On average, they displayed a significant goal attainment over the course of time (d = 0.75; see Table 7). Effect size was medium for goals that were classified as “developing a resource” (d = 0.66) and large for goals that were classified as “reducing a problem” (d = 0.84). There was a significant but small correlation between the extent of changes in individual goal behavior and the duration between the initial and last measurements, with more improvement after a longer time interval (r = 0.15, p < .0001).

**Table 7:** Most frequently used goal categories and changes in individual goals from the initial to last measurement

**N = 433**	**Number of goals**	**M(t1)**	**M(tn)**	**SD (t1)**	**SD (tn)**	**t**	**df**	**p**	**Effect size**	**Improvement (%)**
Progress at school	193	3.86	4.69	1.36	1.63	7.91*	192	<.0001	0.55	54.4
Progress and behavior at vocational training	181	3.63	4.76	1.59	1.71	8.55*	180	<.0001	0.68	60.8
Behavior at school	156	3.35	4.56	1.27	1.63	9.74*	155	<.0001	0.83	63.5
Leisure activities	121	3.28	4.12	1.55	2.02	4.78*	120	<.0001	0.47	52.1
Independence	120	3.63	4.58	1.28	1.52	6.83*	119	<.0001	0.68	56.7
Health behavior	120	3.36	4.53	1.44	1.59	7.15*	119	<.0001	0.77	59.2
Social competence	69	3.52	4.83	1.21	1.46	7.14*	68	<.0001	0.98	75.4
Relationship to family members	65	3.15	4.45	1.27	1.49	7.01*	64	<.0001	0.94	64.6
Management of conflicts / ability to criticize	57	3.21	4.35	1.31	1.43	8.13*	56	<.0001	0.83	71.9
Reliability / responsibility	49	3.92	5.20	1.48	1.59	6.00*	48	<.0001	0.83	57.1
Other	363	3.22	4.56	1.37	1.64	16.74*	362	<.0001	0.89	66.7
All individual goals	1494	3.45	4.59	1.41	1.65	27.49*	1493	<.0001	0.75	61.6

Effect size = Cohen’s d: 0.5-0.8 medium effect, > 0.8 large effect; improvement = percentage of clients who showed increase in this goal behavior; p-level (adjusted) for 12 t-Tests: *p = .01 → p = .00083.

Furthermore, individual goals were classified by their titles into 20 categories to allow more detailed analyses. Table 7 shows the ten most frequently used goal categories. The goals that were set most often involved behavior and progress in school and vocational training (35.5% of all goals). Depending on the goal category, children and adolescents exhibited improvement in 52% – 75% of the individual goals. The biggest changes occurred for goals having to do with “social competencies” (d = 0.98) and “relationship to family members” (d = 0.94).

### Changes in social goals in relation to changes in mental health and behavior problems

The magnitude of changes in goal behavior differed in relation to changes in mental health and behavior problems in the following four domains: future perspective, conflict management, ability to criticize / take criticism, and reliability / rule compliance. Effect sizes ranged from small to medium (see Table 8). The greatest increase in goal behavior occurred in all eight domains if mental health and behavior problems decreased over time (T ≥ 60 → T < 60). In cases where behavior problems became (borderline) clinical while in child welfare services (T < 60 → T ≥ 60), mean goal behavior decreased in six of the domains, but the changes were not significant. In addition, correlations between the extent of changes in social goals and in emotional and behavioral problems (broadband scales of CBCL & YSR) were analyzed. The magnitude of changes in seven goal behaviors correlated with changes in the CBCL total score, while none correlated with the CBCL internalizing problem scale. Changes in the YSR total score correlated with changes in four goal behaviors (see Table 9).

**Table 8:** Changes in social goals from the initial to last measurement in relation to changes in mental health and behavior problems (CBCL total problems)

**N = 329**	**T < 60** → **T < 60 (N = 80)**^**#**^	**T ≥ 60** → **T < 60 (N = 71)**	**T < 60** → **T ≥ 60 (N = 31)**	**T ≥ 60** → **T ≥ 60 (N = 147)**	**F**	**df**	**p**	**Effect size**
Independence	0.33	0.72	0.32	0.61	1.66	3/325	.1750	0.12
Future perspective	0.00	1.23	−0.48	0.29	10.78*	3/325	<.0001	0.32
Conflict management	0.36	1.23	−0.06	0.59	8.76*	3/325	<.0001	0.28
Ability to criticize / take criticism	0.08	1.01	−0.29	0.50	8.33*	3/325	<.0001	0.28
Reliability / rule compliance	0.00	0.77	−0.23	0.10	6.19*	3/325	.0004	0.24
Behavior at school / vocational training	0.08	0.63	−0.29	0.44	2.73	3/325	.0439	0.16
Integration into (peer)groups / friendship	0.44	1.01	0.16	0.47	3.76	3/325	.0111	0.19
Ability to communicate	0.26	0.73	−0.06	0.40	3.56	3/325	.0147	0.18
Total score	0.19	0.92	−0.12	0.43	12.27*	3/325	<.0001	0.34

CBCL = Child Behavior Checklist/4-18; effect size = Cohen’s f: 0.1-0.25 small effect, 0.25-0.4 medium effect; p-level (adjusted) for 9 ANOVAs: *p = .01 → p = .0011; ^#^ For further explanations regarding group definition, see section Statistical analyses.

**Table 9:** Correlation matrix between changes in social goals and changes in CBCL/YSR from the initial to last measurement

	**CBCL internalizing**	**CBCL externalizing**	**CBCL total problems**	**YSR internalizing**	**YSR externalizing**	**YSR total problems**
**(1) Ability to communicate**	−0.12	−0.23*	−0.24*	−0.20	−0.20	−0.21*
**(2) Conflict management**	−0.13	−0.30*	−0.29*	−0.19	−0.17	−0.21
**(3) Ability to criticize/ take criticism**	−0.18	−0.19	−0.24*	−0.19	−0.16	−0.20
**(4) Reliability/ rule compliance**	−0.17	−0.28*	−0.28*	−0.14	−0.23*	−0.23*
**(5) Independence**	−0.07	−0.13	−0.16	−0.22*	−0.16	−0.21*
**(6) Behavior at school/ vocational training**	−0.07	−0.25*	−0.23*	−0.16	−0.26*	−0.25*
**(7) Future perspective**	−0.10	−0.25*	−0.24*	−0.17	−0.20	−0.18
**(8) Integration into (peer)groups/ friendship**	−0.15	−0.21*	−0.22*	−0.23*	−0.09	−0.19
**(9) Total score**	−0.19	−0.36*	−0.36*	−0.28*	−0.29*	−0.32*
**(10) Individual goals**	−0.10	−0.14*	−0.15*	−0.07	−0.12*	−0.09

Correlations were based on N = 329 for social goals*CBCL and N = 313 for social goals*YSR; N = 1085 for individual goals*CBCL and N = 1027 for individual goals*YSR; Pearson’s correlation coefficient r: 0.1-0.3 small effect, 0.3-0.5 medium effect, > 0.5 large effect; p-level (adjusted) for 60 correlations: *p = .01 → p = .000167.

### Changes in individual goals in relation to changes in mental health and behavior problems

The magnitude of changes in individual goals did not differ significantly between young people for whom the extent of emotional and behavioral problems changed over time and for those with a constant high or low level of problems (ANOVA: F = 1.53, df = 3/1081, p = .206, Cohen’s f = 0.07). However, there were negative correlations between changes in those instruments, meaning that improvement in individual goals was to some extent linked to a decrease of externalizing behavior (see Table 9).

## Discussion

In our study, competencies and problem behavior of children and adolescents residing in youth welfare institutions (day care and residential care) as well as changes in their behavior were measured. As expected, at the beginning young people often exhibited impaired quality of life and mental health and behavior problems. Large-scale studies in Great Britain, the US and Germany showed that there were considerable mental health needs in young people in welfare institutions as well [8,15,16]. Thus the participants in our study were comparable to those in other large-scale studies involving mental health and behavior problems.

### How do mental health and behavior problems and the social behavior of children and adolescents change while they are receiving youth welfare services?

In the youth welfare studies mentioned above, about two-third of the children and adolescents showed overall improvement while in care [53,54]. In our study, mental health and behavior problems decreased significantly for young people receiving youth welfare services from the adolescents’ as well as the caregivers’ point of view. However, we do not know if this decrease is a result of the environment inside the day care and residential care institutions or if this decrease occurs in relation to other changes in the children’s and adolescents’ lives, e.g. changes having to do with their parents and friends or with usual child development.

Moreover, young people exhibited significantly more socially competent behavior after receiving youth welfare services. The greatest progress was in the areas of “independence” and “management of conflicts”. In four of the eight social goals, young people showed more improvement after a prolonged stay in youth welfare, implying that a longer and thus more expensive care may be worthwhile. Even so, social competencies usually increase with age. Therefore, a comparison with young people who do not receive youth welfare services is needed in order to estimate how much of the improvement may be due to general child development.

In addition, young people with a lack of competent behavior were found to improve more than children and adolescents who were fairly competent to begin with. This could be due to a ceiling effect of the goal scale or a regression to the mean over time. However, even smaller changes of this kind still represented improvement, which goes against the apprehension that young people with competent behavior will imitate the poorer behavior of their fellow youth welfare recipients. Our results so far are similar to those found in other youth welfare studies [53], both using internationally established screening instruments (CBCL, YSR) as well as using a newly developed tool for youth welfare contexts (social goals).

### To what extent do children and adolescents achieve individual goals while receiving youth welfare services? And which topics are frequently represented in these individual goals?

It was particularly in goals involving individual social competencies that young people showed significant improvement in individual goal behavior. Most often, the goals that were set had to do with progress and behavior in school or vocational training. Effect sizes were larger than for social goals and problem behavior. This confirms earlier findings: that individual goal attainment scaling is more sensitive to individual change than are standardized questionnaires and global measurements [34,36].

While behavior improved in most of the individual goals, the targeted characteristic was only met in about one third of the cases. It seems like the goals were not realistic, suggesting that caregivers need to be trained how to set achievable goals.

In the JES study, more than half of the goals were reached [53]. Comparing this to our findings, however, is difficult, since goal attainment was rated by the professionals alone, whereas in our study, caregivers and young people rated the goals cooperatively, thereby meeting a requirement of the “German law to improve protection of children and adolescents” with regard to enabling young people to participate [55].

### How do changes in social and individual goals relate to changes in mental health and behavior problems?

Children and adolescents whose mental health and behavior problems decreased over time exhibited the greatest improvement in social goal behavior. Whenever behavior problems increased, there were no significant changes in social goals. There was no significant difference in individual goal attainment regardless of whether mental health and behavior problems of children and adolescents decreased, remained constant, or increased. Individual goal behavior improved under all conditions. Correlations between changes in those instruments, however, suggested that improvement was greater if the problems - especially those involving externalizing behavior - decreased. Considering that individual goals may refer to all kinds of youth behaviors and not only to mental health and behavior problems recorded by CBCL, these findings are in accord with assumptions about goal attainment scaling, namely that it is a flexible instrument that is able to measure even small, but relevant, changes [34].

### Limitations

There are some limitations that need to be mentioned. In this study, a variety of services in the heterogeneous German youth welfare system were analyzed together. There may well be differences between institutions as well as types of treatments that were not examined. Furthermore, there was no control group to compare our findings with, and thus we cannot know how much of the behavioral change might be due to the typical maturation of young people. Another aspect that should be pointed out is that the evaluations were performed by caregivers and young people, who may have overestimated the progress of the children and adolescents, instead of by a non-participating third-party rater. However, it was not feasible in this naturalistic study to also use uninvolved evaluators. A further consequence of the naturalistic study design is the wide range of time intervals between measurements as well as missing data. While a more restrictive study design could eliminate these problems, it would not portray youth welfare routines as accurately.

## Conclusions

The current study shows that social and other competencies of children and adolescents increased and emotional and behavioral problems decreased during their stay in day care or residential care institutions. It was especially those with deficits in social competencies who exhibited improvement. Despite ongoing discussions about the high financial costs of youth welfare services, and of residential care in particular, the efficacy of these services cannot be questioned [5,6,38]. One of the reasons for these costs may well be the problems of the clientele who need specialized caregivers and psychosocial care [56].

Since the participation of clients and transparency of services are required by law [3,21,57], instruments meeting these requirements are to be used. Our social and individual goal attainment scales fulfill the prerequisites of social work professionals (strength-based as opposed to psychopathological concepts, sensitivity to individual change) and thus constitute an important addition to established instruments.

## Competing interests

The authors declare that they have no competing interests.

## Authors’ contributions

RK analyzed and interpreted the data and drafted the manuscript. KL designed the study and helped to draft the manuscript. JMF and FK raised the third party funds and helped design the study and to draft the manuscript. MK helped to draft the manuscript. All authors read and approved the final manuscript.
